# Hepatitis C in Pakistan: A Review of Available Data

**Published:** 2010-09-01

**Authors:** Muhammad Umar, Hamama tul Bushra, Masood Ahmad, Available Data, Masood Ahmad, Muhammad Khurram, Saima Usman, Mohammad Arif, Tashfeen Adam, Zahid Minhas, Adnan Arif, Abdul Naeem, Khushnud Ejaz, Zameer Butt, Muhammad Bilal

**Affiliations:** 1 Department of Medicine, Rawalpindi Medical College, Holy Family Hospital, Rawalpindi, Pakistan

**Keywords:** Hepatitis C Virus, Anti-HCV, Pakistan, Serofrequency

## Abstract

Hepatitis C virus (HCV) infection is increasingly recognized as a major health care problem, and is found frequently in Pakistani settings. In this article we reviewed published and unpublished data related to the seroepidemiology of HCV infection in Pakistan. For this article, data from 132 published studies and three unpublished data sets published/ presented between the period 1992-2008 were utilized. Data of 1,183,329 individuals were gathered. Blood donors (982,481) and the general population (178,322) constituted the majority of these subjects. The frequency of HCV infection in blood donors and in the general population was 3.0 % (95% CI: 3.0- 3.1) and 4.7 (95% CI: 4.6 -4.8), respectively. The frequency among 6,148 pregnant females was 7.3% (95% CI = 6.7 – 8.0). The frequency in healthy children ranged from 0.4 to 4.1% (95% CI = 1.4 – 2.3). Pakistani HCV serofrequency figures are significantly higher (P < 0.0001) compared to those of the corresponding populations in surrounding countries like India, Nepal, Myanmar, Iran and Afghanistan.

## Introduction

The World Health Organization (WHO) has compared hepatitis C to a "viral time bomb" and estimates that about 180 million people (some 3% of the world's population) are infected with hepatitis C virus (HCV), 130 million of whom are chronic carriers at risk of developing liver cirrhosis and/or liver cancer. Three to four million persons are newly infected each year, 70% of whom will develop chronic hepatitis. HCV is responsible for 50-76% of all liver cancer cases, and two thirds of all liver transplants in the developed world [1]. World Health Statistics 2008 lists cirrhosis of the liver as the 18th commonest cause of mortality in the world, and it is estimated that by 2030, liver cancer will become the 13th commonest cause [2].

The prevalence of hepatitis varies from country to country, and at times it will also vary among different regions of the same country. The epidemiological estimates by WHO show that the prevalence of hepatitis C is low (< 1%) in Australia, Canada and northern Europe, and about 1% in countries of medium endemicity, such as the USA and most of Europe. It is high (>2%) in many countries of Africa, Latin America, Central and South-East Asia. In these countries, prevalence figures between 5% and 10% are frequently reported [1].

Collecting and comparing health data from across a country is a way to describe health problems, identify trends and help decision-makers set priorities. The global epidemiology of hepatitis C is well established. However, its epidemiology in Pakistan is ill-defined. Most of the data have come from hospital-based studies, because there is a dearth of community-based ones [3][4][5]. Although the National Survey of Hepatitis has concluded, its results have not yet been officially published, except for a few presentations made by researchers at official meetings. This review summarizes the available data on the epidemiology of hepatitis C since the first report of its recognition in 1992.

## HCV-Related Pakistani Data

All available Pakistani data published or unpublished till the writing of this article were collected by a literature search through electronic databases like Pubmed, Pakmedinet, Yahoo and Google etc. Unpublished data from any source, if accessible, were also reviewed. Data from 132 published studies and 3 unpublished data sets were gathered and grouped in six categories, based on the type of population studied i.e. 1) general population, 2) blood donors, 3) patients with liver diseases, 4) patients with diseases other than hepatic diseases, 5) pregnant women and 6) children. Most of the data (78.5%) pertained to the years 2001-2008. Year by year distribution of this data is given in Table 1.

**Table 1:** Distribution of studies and unpublished data sets by year of publication/data gathering/presentation.

**Year**	**Number**	**Percent**
2005 - 2008	47	34.8
2001 - 2004	59	43.7
1996 - 2000	20	14.8
1995 and earlier.	9	6.7
**Total **	**135**	**100.0**

## General Population

Twenty-five studies [6][7][8][9][10][11][12][13][14][15][16][17][18][19][20][21][22][23][24][25][26][27][28][29][30] pertained to the serofrequency of hepatitis C in the general population. Details are given in Table 2 The majority of these studies (92%) were conducted during the period 2000-2008. Two studies were conducted in the nineties [6][7]. The data of 178,322 persons were included in this group. Unfortunately, there was no study from any major city of Balochistan, or from the NWFP provinces. The frequency of HCV infection ranged from 0.4% in Karachi [6] to 33.7% in Jarwar (Sindh) [28]. The mean frequency was 4.7% (95% confidence interval [CI]: 4.6 - 4.8).

**Table 2:** Sero-Prevalence of HCV in General Population

**Author**	**Year**	**Place**	**Number**	**Anti HCV (%)**	**Reference**
Agboatwala *et al.*	1994	Karachi	258	0.4	[6]
Luby	1997	Hafizabad	313	6.5	[7]
Aslam	2001	Lahore	488	16.0	[8]
Aslam	2001	Gujranwala	1,922	23.8	[8]
Ali *et al.*	2002	Rawalpindi	5,370	3.3	[9]
Khan	2004	Mardan	700	9.0	[10]
Khokhar	2004	Islamabad	47,538	5.3	[11]
Muhammad	2005	Buner	16,400	4.6	[12]
Farooq *et al.*	2005	Khuzdar	665	3.3	[13]
Hashim *et al*	2005	Attock	4,552	4.0	[14]
Ahmad	2006	Swat	41,613	2.2	[15]
Fayyaz *et al.*	2006	Bahawalpur	2,086	6.3	[16]
Tariq and Janjua	2006	Rawalpindi	15,550	3.7	[17]
Jafri *et al.*	2006	Karachi	3,533	1.6	[18]
Sherif and Tariq	2006	Rawalpindi	2,558	2.8	[19]
Zaman	2006	Bahawalpur	6,815	4.4	[30]
Ahmad *et al.*	2007	Faisalabad	300	16.0	[20]
Altaf *et al.*	2007	Hyderabad	2,835	5.2	[21]
Mirza *et al.*	2007	Bahawalpur	1821	2.5	[22]
Malik *et al.*	2008	Pannu Aqil	5,237	4.8	[23]
Hakim *et al.*	2007	Karachi	4,000	5.2	[24]
Buttand Amin	2008	DI Khan	5,707	1.7	[25]
Khan *et al.*	2008	Azad Kashmir	245	3.3	[26]
Idrees *et al.*	2008	Lahore	6,817	14.6	[27]
Abbas *et al.*	2008	Jarwar (Sindh)	873	33.7	[28]
Alam *et al.*	2008	Mianwali	697	1.9	[29]
****	**Total**	****	**178,322**	**4.7 (95% CI: 4.6 -4.8) 6.3)**	

## Blood Donors

Blood donor data constituted the largest data set. There were 27 studies and 3 unpublished data sets in this group. The total population covered in this group was 982,481 over the period 1996-2008 (Table 3). The studies covered all provinces of Pakistan. The serofrequency of HCV ranged from 0.3% in Multan [31] to 12.5% in Islamabad [32]. The mean frequency was 3.0% (95% CI: 3.0- 3.1). Only five studies [29][32][33][34][35] showed a frequency of > 5%.

**Table 3:** Sero-prevalence of HCV in Blood Donors

** Author **	** Year **	** Place **	** Number **	** Anti HCV (%) **	** Reference **
Kakepoto *et al.*	1996	Karachi	16,705	1.2	[103]
Bhatti *et al.*	1996	Rawalpindi	760	4.8	[104]
Lone *et al.*	1999	Lahore	186	4.3	[105]
Mujeeb	2000	Karachi	612	0.5	[106]
Tanwani and Ahmad	2000	Islamabad	1,345	12.5	[32]
DBTU	2001 UP	Rawalpindi	20,500	5.0	[99]
PBTS	2001 UP	Lahore	120,000	2.3	[99]
Adridi	2001 UP	Peshawar	19,000	4.0	[99]
Ryas *et al.*	2001	Rawalpindi	1,885	4.7	[107]
Ahmed *et al.*	2001	Karachi	1,410	4.4	[108]
Ahmad *et al.*	2002	Lahore	5,789	4.9	[109]
Khattak *et al.*	2002	Rawalpindi	103,858	4.0	[110]
Fayyaz *et al.*	2002	Bahawalpur	345	5.6	[33]
Mumtaz *et al.*	2002	Rawalpindi	553	6.2	[34]
Rahman *et al.*	2002	Lahore	166,183	4.4	[111]
Ali *et al.*	2003	Quetta	1635	1.8	[112]
Akhtar *et al.*	2004	Karachi	351,309	1.8	[113]
Ahmad *et al.*	2004	Peshawar	4,000	2.2	[114]
Mahmood *et al.*	2004	Multan	6,000	0.3	[31]
Sirhindi	2006	Lahore	18,216	4.2	[115]
Aziz	2006	Skardu	850	1.1	[116]
Khan	2006	Bahawalpur	27,938	2.5	[117]
Ujjan *et al.*	2006	Hyderabad	3,677	6.7	[35]
Mujeeb *et al.*	2006	Karachi	7,325	3.6	[118]
Chaudhary	2007	Rawalpindi	1,428	2.5	[119]
Alam and Naeem	2007	Gilgit	8.949	1.3	[120]
Ishaq *et al.*	2007	Thatta	310	1.3	[121]
Bhatti *et al.*	2007	Karachi	94,177	4.2	[122]
Khattak *et al.*	2008	Peshawar	1,131	4.1	[123]
Mujeeb and Pearce	2008	Karachi	5,345	7.5	[124]
****	**Total**		**982,481**	**3.03 (95% CI: 3.0- 3.1)**	

## Patients with Liver Diseases

Forty-one studies [36][37][38][39][40][41][42][43][44][45][46][47][48][49][50][51][52][53][54][55][56][57][58][59][60][61][62][63][64][65][66][67][68] dealing with the frequency of hepatitis C among patients with liver diseases were found, covering 7,765 patients over a period of 27 years from 1992 to 2008 (Table 4,Table 5). Liver diseases included cirrhosis, chronic liver disease, chronic active hepatitis and hepatocellular carcinoma.

Fourteen studies [37][38][39][40][41][42][43][44][45][46][47][48][49][50] pertained to cirrhosis patients. The number of individuals included in these studies was 1,902. HCV serofrequency in these individuals ranged from 10.5-79.6%, with an overall prevalence of 44.9% (95% CI = 42.6 - 47.2). Eleven studies [39][51][52][53][54][55][56][57][58][59][69] covering 3,144 patients with chronic liver disease showed a range of 13.3% to 86.0% HCV serofrequency. The mean frequency was 52.9% (95% CI: 51.1-54.7).

In 10 studies [36][39][40][42][60][61][62][63][64][65] The HCV serofrequency in 739 cases of hepatocellular carcinoma was investigated (Table 5), and it ranged from 24% to 67.9%. The mean frequency in this subgroup was 50.6% (95% CI : 46.9-54.3). One thousand nine hundred and eighty patients with acute/chronic hepatitis from 6 studies had 58.2% (95% CI : 55.7- 60.4) HCV serofrequency [46][51][57][66][67][68].

**Table 4:** Sero-Prevalence of HCV in Liver Disease

**Author**	**Year**	**Place**	**Number**	**Anti HCV (%)**	**Reference**
**Cirrhosis**					
Malik	1992	Northern Areas	24	13.3	[38]
Malik	1995	Rawalpindi	220	48.0	[39]
Makki	1995	Karachi	67	10.5	[40]
Hussain	1998	Lahore	50	52.0	[14]
Farooqi and Farooqi	1998	Peshawar	410	43.9	[42]
Umar *et al.*	1999	Rawalpindi	120	58.0	[43]
Mahmood *et al.*	1999	Karachi	202	37.7	[44]
Liaqat	2000	Karachi	250	43.0	[45]
Shah *et al.*	2002	Islamabad	108	79.6	[46]
Iqbal	2002	Peshawar	100	41.0	[47]
Khan	2002	Lahore	94	68.0	[56]
Farooqi and Kahan	2002	Swat	55	59.0	[49]
Khurram	2003	Rawalpindi	142	34.1	[50]
Mashud *et al.*	2004	DI Khan	60	13.3	[125]
****	**Total**	****	**1902**	**44.9 (95% CI = 42.6 - 47.2)**	
**Chronic Liver Disease**					
Malik	1995	Rawalpindi	45	13.3	[39]
Tong *et al.*	1996	Rawalpindi	105	22.0	[52]
Sultana *et al.*	2000	Islamabad	108	29.6	[53]
Younas *et al.*	2001	Lahore	100	65.0	[94]
Tahir *et al.*	2001	Lahore	104	43.3	[55]
Khan *et al.*	2001	Islamabad	323	28.7	[56]
Khokhar	2002	Islamabad	354	86.0	[51]
Khan	2003	Hazara	614	40.8	[57]
Shaikh	2003	Larkana	1,074	51.0	[69]
Bakhtiari *et al.*	2003	Rawalpindi	97	68.1	[58]
Wazir *et al.*	2003	Hyderabad	510	59.0	[59]
****	**Total**	****	**3144**	**52.9 ( 95% CI : 51.1 - 54.7)**	

**Table 5:** Sero-Prevalence of HCV in Hepato-cellular Carcinoma and Acute/Chronic Hepatitis

**Author**		**Year**		**Place**		**Number**		**Anti HCV (%)**		**Reference**
**Hepato cellular Carcinoma**										
Makki	1991		Karachi		25		24.0		[40]	
Malik	1995		Rawalpindi		64		25.0		[39]	
Mujeeb *et al.*	1997		Karachi		54		33.0		[60]	
Farooqi and Farooqi	2000		Peshawar		56		67.9		[42]	
Mumtaz *et al.*	2001		Rawalpindi		44		54.0		[61]	
Khokhar *et al.*	2003		Islamabad		67		67.0		[72]	
Siddiqui	2005		Karachi		100		43.0		[36]	
Bilal	2006		Faisalabad		100		66.4		[45]	
Almani *et al.*	2008		Hyderabad		100		52.0		[64]	
Abbas *et al.*	2008		Karachi		129		51.2		[65]	
****	**Total**		****		**739**		**50.6 (95% CI =46.9 - 54.3)**			
**Acute /Chronic Hepatitis**										
Haider *et al.*	1994		Lahore		94		6.4		[79]	
Umer	1999		Rawalpindi		710		70.0		[67]	
Almani *et al.*	2002		Jamshoro		100		9.0		[112]	
Khokhar	2002		Islamabad		354		86.0		[51]	
Shah *et al.*	2002		Islamabad		108		79.6		[115]	
Khan *et al.*	2003		Abbotabad		614		40.8		[57]	
****	**Total**		****		**1980**		**58.2 (95% CI =55.9 - 60.4)**			

## Patients with Diseases Other than Hepatic Diseases

Sixteen studies focused on HCV serofrequency in patients with various diseases other than liver diseases (Table 6) [10][70][71][72][73][74][75][76][77][78][79][80][81][82][83][84][85]. These studies spanned a period of 8 years, from 1999 to 2006, and covered major cities. The mean frequency of HCV infection in patients with various medical diseases was 20.4% (95% CI : 18.4-22.5), 38% in haematological diseases (95% CI : 33.6-42.5), 7.9% among surgical diseases (95% CI : 6.9 - 9.0), and 1.3% in dental diseases.

**Table 6:** Seroprevalence among patients of diseases other than Liver Disease.

** Author **	** Year **	** Place **	** Disease **		** Number **	** Anti HCV (%) **	** Reference **
**Medical Diseases**							
Khan	2004	Mardan	General		700	9.0	[10]
Mumtaz andAftab	2005	Rawalpindi	General		264	28.6	[70]
Gul and Iqbal	2003	Lahore	Renal Failure		50	68.0	[71]
Khokar *et al.*	2005	Islamabad	Renal Failure		97	23.7	[72]
Qazi *et al.*	2004	Bahawalpur	Diabetes 2		250	27.6	[73]
Bilwani *et al.*	2004	Karachi	Lymphoprolifrative		143	29.2	[89]
		**Total **			** 1504 **	** 20.4 (95% CI = 18.4-22.5) **	
** Haematological Diseases **							
Moatter *et al.*	1999	Karachi	β -Thalassemia	100		35.0	[106]
Muhammad J	2003	Peshawar (Thalassemia)	β -Thalassemia	80		36.2	[126]
Younus *et al.*	2004	Islamabad	β -Thalassemia	75		42.0	[94]
Burki *et al.*	2005	Islamabad	β -Thalassemia	180		41.7	[74]
Hussain	2001	Peshawar	Haemophillia	40		25.0	[14]
		**Total **		** 475 **		** 38 (95% CI = 33.6-42.5) **	
**Psychological Diseases **							
Shah and Dar	2004	Islamabad	Depression	135		22.9	[80]
Surgical Diseases							
Hussain and Fatima	2005	Karachi	Surgical Diseases	750		16.2	[81]
Talpur *et al.*	2006	Nawabshah	Surgical Diseases	180		11.9	[82]
Khan *et al.*	2007	Abbottabad	Orthopaedic Diseases	1630		3.1	[83]
Daudpota and Soomro	2008	Jacobabad	Surgical Diseases	150		14.0	[84]
		**Total **		** 2710 **		** 7.9 (95% CI = 6.9-9.0) **	
**Dental Diseases **							
Khitab *et al.*	2005	Peshawar	Dental Diseases Diseases	1,498		1.3	[85]

## Pregnant Women

Eleven studies [86][87][88][89][90][91][92][93][94][95][96] dealing with the serofrequency of HCV among pregnant women were found (Table 7). These studies spanned a period of 13 years from 1996 to 2008 and covered 6,148 women. The frequency in this group ranged from 3.3% to 29.1%. Overall serofrequency in this group was 7.3% (95% CI = 6.7 - 8.0).

**Table 7:** Seroprevalence among Pregnant women

**Author**	** Year **	** Place **	** Number **	** Anti HCV (%) **	** Reference **
**Pregnant Women**					
Khan	1996	Lahore	417	9.3	[56]
Parker	1999	Lahore	417	4.0	[90]
Zafar *et al.*	2001	Lahore	300	6.0	[88]
Bilal	2002	Peshawar	352	5.1	[74]
Rizvi	2002	Karachi	120	6.6	[80]
Fayyaz	2004	Lahore	100	7.0	[91]
Khokar *et al.*	2004	Islamabad	503	4.8	[92]
Jaffery	2005	Islamabad	947	3.3	[93]
Yousafani *et al.*	2006	Hyderabad	103	29.1	[94]
Hakeem *et al.*	2006	RY Khan	450	18.2	[41]
Batool *et al.*	2008	Lahore	2,439	7.3	[99]
	**Total **		** 6,148 **	**7.3 (95% CI = 6.7 - 8.0) **	

## Children

Data from four studies [18][86][97][98] covering 4,472 healthy children were analyzed (Table 8). HCV serofrequency among children ranged from 0.4% to 36.25%. The mean frequency was 1.8% (95% CI = 4.6 - 7.5). One study from Lahore in 1996 reported a 4.1% perinatal transmission of HCV in children of HCV- positive mothers. The serofrequency of HCV in thalassemics, and hemophiliacs was 35-42% [75][76][77][78] and 25% [79], respectively.

**Table 8:** Seroprevalence among Healthy Children

**Author**	**Year**	**Place**	**Number**	**Anti HCV (%)**	**Reference**
Jafri *et al.*	2006	Karachi	3,533	1.6	[18]
Akram	1995	Karachi	230	0.40	[97]
Khan	1996	Lahore	538	4.09	[86]
Hyder	2001	Lahore	171	0.58	[98]
****	**Total**		** 4472 **	** 1.8 (95% CI = 1.4 - 2.3) **	

## Discussion

Many researchers have tried to derive a national or regional figure for HCV serofrequency based on the available data [99][100][101]. In 2005, Umar M and Khaar HTB [99], from Rawalpindi, collected data from various studies and reported a 9.8% frequency in 79,192 individuals. In 2006, Hafeez [99], from Islamabad, collected data from 98 studies and produced an estimate of 5.3%. Raja and Janjua [101], from Essex (UK), analyzed studies available on Medline from 1970 to 2005, and observed that the prevalence of HCV infection ranges from 0.4% in children, to 1.2% in healthy blood donors. Ali et al. reviewed data from 139 studies from 1994 to 2007 and concluded that the weighted average for hepatitis C antibody positivity was 3.0% (range 0.3-31.9%). Rates in the high-risk subgroups were far higher [100].

The data analyzed in this article shows HCV positivity (95% CI: 4.6 - 4.8) among 178,322 healthy individuals and 3.03% positivity (95% CI: 3.00 - 3.06) among 982,481 healthy blood donors. This figure is significantly lower (P < 0.0001) than the figure of 9.8% quoted by Umar M and Khaar HTB [99]. However, our estimates are significantly higher (P < 0.00001) than the 3% figure quoted by Ali et al. [100] for healthy individuals. Our estimates are closer to the figure of 5.3% given by Hafeez [99]. According to several studies, HCV genotype 3 is the most frequent genotype found in Pakistan (Table 9).

Most of the available Pakistani data on HCV pertains to blood donors, probably because for reasons of convenience and access to a large sample size, and may not truly represent the general population [100]. If high-risk blood donors are screened out (those with jaundice, injection drug users etc), the frequency in the general population may be underestimated. If professional blood donors (who are often injection-drug users who sell their blood to make money) are included, the figures may be overestimated.

The number of subjects studied, other than healthy blood donors, appears very small, and also there is a lack of representation from across the country [101]. The frequency of HCV positivity in Pakistan is significantly higher (P < 0.0001) when compared to the corresponding populations in the surrounding countries (Table 10).

We noted a highly variable serofrequency in different studies of similar populations, even within the same province. One of the reasons for such variability in the results has been explained by Ali et al. [100]. The authors observed that unlike highly contagious diseases like measles that have a more predictable seroprevalence, blood-borne illnesses like hepatitis and HIV are transmitted sporadically or in microepidemics. According to them these microepidemics may account for wide variations in prevalence within a nation, a province, or even a community, and methodological differences in sampling strategies may also contribute to differences in seroprevalence within similar regions or populations [100].

According to the "Burden of Disease Study" carried out by Hyder and Morrow in 2001, chronic liver diseases are the 5th commonest cause of premature mortality in Pakistan and the 11th commonest cause of disabilities [102]. Although between 75-85% of infections move on to chronic hepatitis C, the progress may be slow. Hence most people who are infected do not experience symptoms and are unaware of their infection. They are not able to benefit from available treatment that may clear them of the virus. They may also unknowingly spread the virus to others. Currently, there is no vaccine to prevent HCV infection. Effective but costly treatment is available. The high frequency and its contribution to premature mortality and disability call for massive awareness campaigns to combat the menace of this infectious disease.

**Table 9:** Frequency of Genotypes^a^

**Author**	**Year**	**Place**	**Number (Typable)**	**Genotype (%)**							**Reference**
				**1**	**2**	**3**	**4**	**5**	**6**	**Mixed**	
Azhar	2003	Bahawalpur	105	6.0	4.8	69.6	2.4	-	0.8	-	[122]
Ansari	2002	Karachi	255	12.0	2.3	77.6	2.3	0.4	2.7	2.3	[127]
Khokhar	2002	Islamabad	148	-	-	64.1	-	-	-	-	[125]
Zuberi	2002	Karachi	215	8.4	-	79.5	-	-	-	8.8	[88]
Nasir	2001	Rawalpindi	39	5.1	2.6	59.9	5.2	12.8	15.4	-	[128]
Mumtaz	2005	Karachi	725	7.9	1.2	87.8	5.0	-	0.8	-	[129]
	**Total**		**725**	**7.7**	**1.6**	**80.5**	**3.1**	**0.9**	**1.3**	**1.7**	

^a^ Due to missing data in some studies, percentages do not add to 100

**Table 10:** Comparison of HCV Frequency with surrounding countries

**Country**	**Year**	**Population**	**Number**	**Prevalence (%)**	**Reference**
Myanmar	2006	Healthy Adults	362	2.5	[130]
India	2003	Blood Donors	28,956	0.66	[126]
Nepal	2004	Healthy Adults	103	1.0	[131]
Iran	2006	Healthy Adults	1721	1.0	[132]
Afghanistan	2006	Healthy Women	4452	0.31	[133]
Pakistan	2009	Healthy Adults	178,322	4.7	This review

## Conclusions

The frequency of hepatitis C infection in Pakistan is high (4.7%), varying from 0.4% - 33.7%, indicating pockets of infections. The frequency is significantly higher than in surrounding countries. The contribution of this high frequency to premature mortality and disability calls for massive awareness campaigns.
